# *Cudrania tricuspidata* water extract improved obesity-induced hepatic insulin resistance in *db/db* mice by suppressing ER stress and inflammation

**DOI:** 10.3402/fnr.v59.29165

**Published:** 2015-10-26

**Authors:** Ok-Kyung Kim, Da-Eun Nam, Woojin Jun, Jeongmin Lee

**Affiliations:** 1 Department of Medical Nutrition, Graduate School of East-West Science, Kyung Hee University, Yongin, Republic of Korea; 2 Division of Food and Nutrition, Chonnam National University, Gwangju, Republic of Korea

**Keywords:** *Cudrania tricuspidata*, obesity, insulin resistance, ER stress, inflammation

## Abstract

**Background:**

Obesity can play a role in the development of hepatic insulin resistance. Although the molecular mechanism of the association between obesity and hepatic insulin resistance is unclear, it has been reported that obesity leads to hepatic endoplasmic reticulum (ER) stress and inflammation, which can induce the development of insulin resistance in several tissues.

**Objective:**

In this study, we investigated the associations between hepatic insulin resistance, ER, and inflammation in obesity and the effect of water extract from *Cudrania tricuspidata* leaves (CTL) on hepatic insulin resistance induced by ER stress and inflammation in *db/db* mice.

**Design:**

The mice were randomly divided into four groups: a normal control group (C57BL/6J), a control group (C57BL/6J-*db/db*), a CTL 100 group (C57BL/6J-*db/db* mice fed a dietary supplement of 100 mg/kg of CTL), and a CTL 300 group (C57BL/6J-*db/db* mice fed a dietary supplement of 300 mg/kg of CTL). After 8 weeks, we performed an oral glucose tolerance test and the mice were sacrificed.

**Results:**

The C57BL/6J-*db/db* mice developed obesity and hyperglycemia, and the ER stress response and inflammation were activated in their livers. Interestingly, there was a marked decrease in the activation of the ER stress response and insulin resistance in the livers of the C57BL/6J-*db/db* mice treated with CTL due to decreases in the phosphorylation of eIF2α, IRE1α, and IRS-1 serine and decreases in the mRNA expression of ATF4, c-Jun N-terminal kinase, C/EBPα, and C/EBP homologous protein. Dietary supplementation with CTL also induced a statistically significant decrease in the expression of pro-inflammatory cytokines, C-reactive protein (CRP), and NF-κB phosphorylation.

**Conclusions:**

Overall, these results suggest that CTL can improve hepatic insulin resistance and hyperglycemia by controlling obesity-induced ER stress and inflammation in the liver and that CTL may be a useful agent in treating hyperglycemia.

The liver plays a crucial role in the regulation of glucose homeostasis, which is tightly regulated by several hormones and enzymes during post-absorptive and fasting periods (1–3). Insulin is a key regulator of glucose metabolism, with the hormone directly and indirectly regulating glucose metabolism by binding to insulin receptors (IRs) in the liver (4, 5). During normal insulin action in the liver, insulin binds to IRs and induces tyrosine phosphorylation of IR substrate (IRS) proteins. IRS tyrosine phosphorylation causes the activation of phosphatidylinositol-3-kinase (P13-K), which results in the inhibition of the transcription of gluconeogenic enzymes, including phosphoenolpyruvate carboxykinase (PEPCK) and glucose-6-phosphatase (G6Pase) (6–8). In contrast, during fasting periods, gluconeogenesis is strongly stimulated by low levels of insulin and high levels of glucagon (3, 9, 10). Abnormal insulin action in the liver results in insulin resistance characterized by an impaired ability of insulin to inhibit glucose output, leading to gluconeogenesis and hyperglycemia (11, 12).

Obesity is a major factor in the development of hepatic insulin resistance. The molecular mechanism linking obesity to hepatic insulin resistance is unclear and controversial (13–15). Recent studies reported that inflammatory signals, including those produced by excess lipids, in an obese state can stimulate endoplasmic reticulum (ER) stress and inflammation in several cells and can play key roles in insulin resistance (11, 12, 16).

In ER stress, three ER transmembrane proteins are activated: PKR-like endoplasmic reticulum kinase (PERK), activating transcription factor 6 (ATF6), and inositol-requiring enzyme 1 (IRE1). Under normal conditions, these three ER transmembrane proteins are maintained in an inactive state by binding to the binding immunoglobulin protein, a chaperone protein also known as ‘78 kDa glucose-regulated protein’ (GRP78). The dissociation of the chaperone protein from each ER transmembrane protein during the folding of unfolded proteins can trigger their activation and the induction of the ER stress response (17–19). ER stress induces the activation of the c-Jun N-terminal kinase (JNK) pathway, leading to the suppression of the IR and the expression of gluconeogenic enzymes (20). In addition, ER stress activates nuclear factor-κB (NF-κB) signaling, which induces the expression of inflammatory mediators. These inflammatory mediators can also induce ER stress (21, 22). Therefore, chronic obesity induced by chronic excess energy intake leads simultaneously to inflammation-induced ER stress and ER stress-induced inflammation, which further aggravate both ER stress and the inflammation. Taken together, ER stress and inflammatory pathways in obesity directly or indirectly disrupt the metabolic functioning, including glucose and lipid metabolism, of several tissues (20–24).

There is increasing evidence that some natural plants can suppress hyperglycemia. One such plant is *Cudrania tricuspidata* (CT), which is used in Korean folk medicine (25–27). One study reported that CT leaves inhibited lipase activity, reduced plasma triacylglycerol levels, and delayed lipid absorption *in vivo* (28). In addition, recent studies found that CT has anti-inflammation and antihyperglycemia effects (29–31). Although there is increasing evidence of various physiological activities by CT, the detailed molecular mechanisms for these activities have not yet been elucidated. Findings on the physiological activity of CT suggest that it might improve obesity-induced hepatic insulin resistance and hyperglycemia. Therefore, the aim of this study was to investigate the associations among hepatic insulin resistance, ER stress, and inflammation in obesity as well as the effects of *C. tricuspidata* leaf water extract (CTL) on obesity-induced hepatic insulin resistance caused by ER stress and inflammation in *db/db* mice.

## Methods

### *Cudrania tricuspidata* leaves extracts and standardization

*Cudrania tricuspidata* leaves were obtained from the Agriculture Corporation for Goseong and extracted by the Korea INS Pharm Inc. (Chonnam, Korea). The dried leaves (50 g) were heated in distilled water (1 L) at 100°C for 4 h in a reflux apparatus and filtered. The extracts were concentrated *in vacuo* and lyophilized. The dried *Cudrania tricuspidata* leaf water extract (CTL) was obtained at a yield of 15.6% and kept in a tightly sealed, light-protected container at−20°C until used.

### Experimental animals and supplement

The experimental protocols described in this study were approved by the Institutional Animal Care and Use Review Committee of Kyung Hee University [KHUASP(SE)-14-011]. C57BL/6J-*db/db* mice (20–23 g, 5 weeks, male) and age-matched wild-type C57BL/6J mice (20–23 g, 5 weeks, male) were purchased from SLC Inc. (Hamamatsu, Japan). The animals were housed in wire mesh–bottomed individual cages in climate-controlled quarters (24±1°C, 55±5% relative humidity) with a 12-h light:12-h dark cycle. All of the mice were acclimatized for an adaptation period of 7 days before the experiment, fed standard pellet chow, and given fresh water *ad libitum*.

A total of 32 mice were randomly divided into four groups (eight mice per group): a normal control group (C57BL/6J), a control group (C57BL/6J-*db/db*), a CTL 100 group (C57BL/6J-*db/db* mice fed a dietary supplement of 100 mg/kg of CTL), and a CTL 300 group (C57BL/6J-*db/db* mice fed a dietary supplement of 300 mg/kg of CTL). Mice in each group were fed their experimental diets, which were based on the AIN93G diet, for 8 weeks.

### Oral glucose tolerance test

At the end of 8 weeks, mice were subjected to the oral glucose tolerance test (OGTT). The mice were fasted overnight and then 1 g/kg glucose solution was orally administered. Blood samples were obtained by the tail-clip method at various time points (30, 60, and 120 min). Blood glucose levels were determined using the ACCU-CHEK Advantage glucose analyzer (Roche Diagnostics, Basel, Switzerland).

### Serum biomarker analyses

At the end of 8 weeks, all mice were sacrificed by cervical dislocation. Serum was collected from the whole blood by centrifugation (12,000 rpm, 4°C, for 20 min). Serum insulin, glucagon, and leptin were respectively measured with an Ultra Sensitive Mouse Insulin ELISA kit (Crystal Chem, Downers Grove, IL, USA), Glucagon Quantikine ELISA kit (R&D Systems, Minneapolis, MN, USA), and Mouse/Rat Leptin Quantikine ELISA kit (R&D Systems) according to the manufacturer's protocols. The levels of serum glucose, triglyceride, total cholesterol, low-density lipoprotein (LDL)/very low-density lipoprotein (VLDL) cholesterol and high-density lipoprotein (HDL) cholesterol were measured using quantification kits from BioVision (Milpitas, CA, USA).

### Histological observation

All mice were sacrificed by cervical dislocation. The liver and fat were rapidly dissected and subjected to observations of lipid droplets. The liver and fat were fixed in 10% neutral buffered formaldehyde solution overnight and rinsed with phosphate-buffered saline. Tissues were embedded in paraffin and stained with hematoxylin and eosin. Sections were observed under a light microscope.

### Protein extraction and Western blot analysis

The livers of each mouse were homogenized in a CelLytic™ MT cell lysis reagent (Sigma-Aldrich, St. Louis, MO, USA) and centrifuged at 12,000 g for 20 min at 4°C. The protein content of the clear lysates was estimated by the Bradford method using protein assay reagent (Bio-Rad Laboratories, Hercules, CA, USA). Equal amounts (100 µg protein/lane) of total protein were dissolved in NuPAGE^®^ LDS sample buffer 4X (Life Technologies, Gaithersburg, MD, USA). Protein samples were separated on 5% or 10% SDS-polyacrylamide gel and transferred to nitrocellulose membranes (Bio-Rad Laboratories). Membranes were incubated for 1 h in a blocking solution containing 5% nonfat milk in Tris-buffered saline and then incubated for 12 h at 4°C with antiβ-actin (1:1,000), antitotal-eIF2α (1:500), antiphospho-eIF2α (1:500), antitotal-IRE1α (1:1,000, Novus Biologicals, Littleton, CO, USA), antiphospho-IRE1α, antitotal JNK (1:1,000), antiphospho-JNK (1:1,000), antitotal NF-κB (1:1,000), antiphospho-NF-κB (1:1,000), antitotal IRS-1 (1:1,000), and antiphospho-IRS-1 (serine, 1:1,000) antibody. With the exception of antitotal-IRE1α antibody, all antibodies were purchased from Cell Signaling Technology (Beverly, MA, USA). After incubation with the primary antibody, membranes were incubated with a secondary antibody (antirabbit IgG HRP-linked antibody, 1:5,000; Cell Signaling Technology) for 1 h at room temperature. Protein bands were developed using the SuperSignal West Dura extended duration substrate (Pierce, Milwaukee, WI, USA) and visualized with ChemiDoc imaging system from Bio-Rad Laboratories.

### Isolation of total RNA and real-time PCR

The liver tissue was homogenized with rotor-stator homogenizers in the presence of buffer RLT (lysis buffer; Qiagen, Valencia, CA, USA) including 1% β-mercaptoethanol. Total RNA was extracted from liver tissue lysate using the RNeasy Mini kit (Qiagen). Complementary DNA was synthesized from 1 µL purified total RNA in 20 µL of reaction buffer using the iScript™ cDNA synthesis kit (Bio-Rad Laboratories). Real-time polymerase chain reaction (PCR) (Applied Biosystems, Foster City, CA, USA) was performed on triplicate samples using 1 µL cDNA with the SYBR Green PCR Master Mix (iQ SYBR Green Supermix, Bio-Rad Laboratories). The cDNA was amplified for 40 cycles of denaturation (95°C for 30 s), annealing (58°C for 30 s) and extension (72°C for 45 s) with specific primers (Table 1). The real-time RT-PCR results were visualized and the relative quantitation was calculated using the 7500 System SDS software version 1.3.1 (Applied Biosystems).

**Table 1:** Primer sets used for real-time polymerase chain reaction (PCR)

Gene	Accession number		Sequence
*GAPDH* (M)	AY618199	F	5′- CAT GGC CTT CCG TGT TCC TA -3′
		R	5′- GCG GCA CGT CAG ATC CA -3′
*TNF-α* (M)	X02611	F	5′- CAC AAG ATG CTG GGA CAG TGA -3′
		R	5′- TCC TTG ATG GTG GTG CAT GA -3′
*IL-1β* (M)	NM008361	F	5′- AGT TGA CGG ACC CCA AAA GA -3′
		R	5′- GGA CAG CCC AGG TCA AAG G -3′
*IL-6* (M)	NM031168	F	5′- CCA CGG CCT TCC CTA CTT C -3′
		R	5′- TTG GGA GTG GTA TCC TCT GTG A -3′
*CRP* (M)	X13588	F	5′- GGT GCT GAA GTA CGA TTC ATG GT -3′
		R	5′- CAG CTG GCA CAG ATG TGT GTT -3′
*ATF4* (M)	NM009716	F	5′- CTC AGA CAG TGA ACC CAA TTG G -3′
		R	5′- GGC AAC CTG GTC GAC TTT TAT T -3′
*XBP-1* (M)	NM013842	F	5′- TGG GCA TCT CAA ACC TGC TT -3′
		R	5′- GCG TCC AGC AGG CAA GA -3′
*CHOP* (M)	X67083	F	5′- AGG AGC CAG GGC CAA CA -3′
		R	5′- TCT GGA GAG CGA GGG CTT T -3′
*GRP78* (M)	AJ002387	F	5′- AGC CAT CCC GTG GCA TAA -3′
		R	5′- GGA CAG CGG CAC CAT AGG -3′
*C/EBPα* (M)	NM001287523	F	5′- GAG CTG AGT GAG GCT CTC ATT CT -3′
		R	5′- TGG GAG GCA GAC GAA AAA AC -3′
*PEPCK* (M)	AF009605	F	5′- CAG CCA CTA GAT TCT GGA TAA CTA TAC AA -3′
		R	5′- TTG CGG GAA ACA AGG ACA AC -3′
*G6Pase* (M)	U00445	F	5′- CAA CCG CCA TGC AAA GG -3′
		R	5′- CTG GCC TCA CAA TGG GTT TC -3′

### Statistical analysis

All experimental data were expressed as mean±standard deviation (SD). The significance of treatment effects was analyzed by Duncan's multiple range tests after one-way ANOVA using SPSS statistical procedures for Windows (SPSS PASW Statistic 20.0, SPSS Inc., Chicago, IL, USA). Statistical significance was considered at the *p*<0.05 level.

## Results

### Effect of CTL on food efficiency rate and organ weights in the C57BL/6J-*db/db* mice

Table 2 shows the weight gain, food efficiency rate (FER), and organ weights. The C57BL/6J-*db/db* mice showed a marked increase in both weight gain and FER compared with the normal mice, indicating that the C57BL/6J-*db/db* mice were obese. Dietary supplementation with CTL significantly reduced the weight gain and FER in a dose-dependent manner. The liver, white adipose tissue, and brown adipose tissue weights of the C57BL/6J-*db/db* control group were significantly higher than those of the normal control group. The liver, white adipose tissue, and brown adipose tissue weights of the CTL dietary supplement groups were significantly decreased compared with those of the C57BL/6J-*db/db* mouse control group. However, there were no significant differences in these parameters between the CTL 100 and CTL 300 groups (*p*<0.05).

**Table 2:** FER and organ weights in the C57BL/6J-*db/db* mice with dietary supplementation of *Cudrania tricuspidata* water extract

		C57BL/6J-*db/db* mice		
		
	Normal control	Control	CTL 100	CTL 300
Initial body weight (g)	23.78±0.50^b^	37.40±1.85^a^	37.03±1.59^a^	37.45±1.12^a^
Final body weight (g)	28.13±0.69^c^	51.98±0.78^a^	49.08±2.02^b^	47.33±2.58^b^
Weight gain (g)	4.35±0.21^d^	14.58±1.27^a^	12.05±0.70^b^	9.88±2.14^c^
FER^1^	3.48±0.17^c^	11.66±1.01^a^	9.64±0.56^b^	7.90±1.71^c^
Organ weight (g/100 g body weight (b.w.))				
Liver	3.44±0.92^c^	7.54±0.84^a^	6.20±0.62^b^	6.22±0.57^b^
White adipose tissue	2.83±1.03^c^	16.51±0.82^a^	14.29±1.29^b^	14.59±1.26^b^
Brown adipose tissue	0.22±0.09^c^	0.45±0.06^b^	0.58±0.08^a^	0.58±0.06^a^

1 FER (food efficiency rate) = weight gain (g)/total food consumption (g)×100.

CTL, water extract from *C. tricuspidata* leaves. All data are expressed as mean±standard deviation (*n*=8). Different letters show a significant difference at *p*<0.05 as determined by Duncan's multiple range test.

### Effect of CTL on OGTT in the C57BL/6J-*db/db* mice

**Fig. 1:**
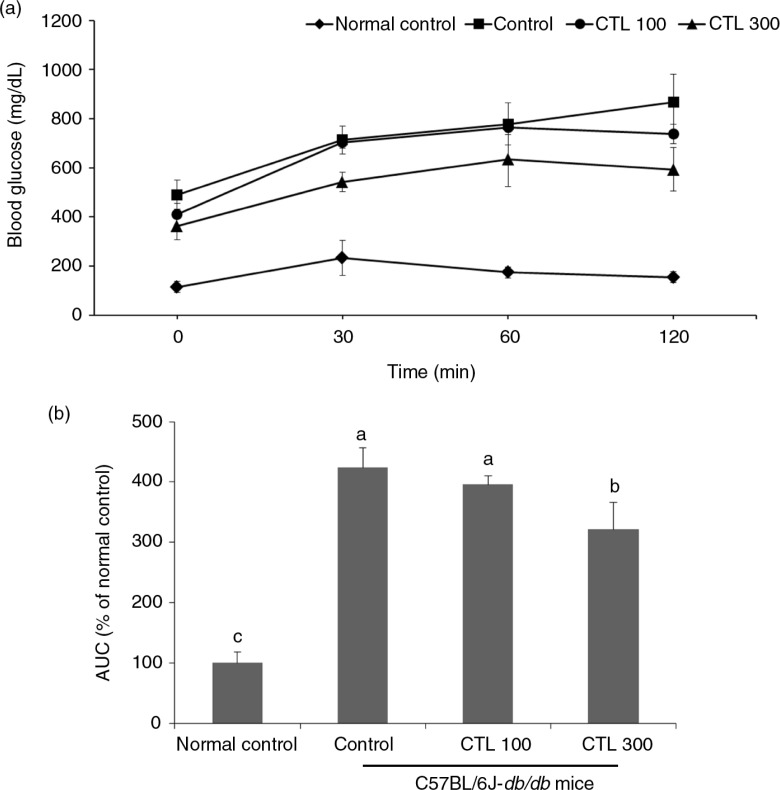
Effect of dietary supplementation with *Cudrania tricuspidata* water extract on oral glucose tolerance test (OGTT) (1 g glucose/kg b.w) in the C57BL/6J-*db/db* mice. (a) Changes in blood glucose during OGTT. (b) Area under the curve (AUC) for OGTT. Data are expressed as mean±standard deviation (*n*=8). Different letters show a significant difference at *p*<0.05 as determined by Duncan's multiple range test.

As illustrated in Fig. 1, there was a considerable increase in the levels of blood glucose at all time points during the OGTT in the C57BL/6J-*db/db* mice compared with the normal mice (Fig. 1a). The area under the curve (AUC) for the blood glucose change in the C57BL/6J-*db/db* mice also increased significantly compared with that of the normal mice. The AUC for glucose increased significantly in the C57BL/6J-*db/db* control group (423.76±32.52; % of the normal control group) compared with that of the normal control group. In the CTL 300 dietary supplement group (321.33±45.25; % of the normal control group), the AUC for glucose decreased significantly compared with that of the C57BL/6J-*db/db* control group, but there was no marked difference between the CTL 100 group and the C57BL/6J-*db/db* control group (Fig. 1b) (*p*<0.05).

### Effect of CTL on levels of glucose, insulin, glucagon, and leptin in the C57BL/6J-*db/db* mice

Fasting glucose and fasting insulin levels were significantly increased in the C57BL/6J-*db/db* mice compared with those of the normal mice. Both the fasting glucose and fasting insulin levels decreased significantly in the CTL dietary supplementation groups compared with those in the C57BL/6J-*db/db* control group. There was no significant between-group difference in these parameters in the CTL 100 and CTL 300 groups. The homeostasis model assessment-estimated insulin resistance (HOMA-IR) of the C57BL/6J-*db/db* control group was significantly elevated (approximately 40-fold) compared to that of the normal control group. The HOMA-IR was decreased significantly in the CTL dietary supplement groups compared with that in the C57BL/6J-*db/db* control group. There were no significant differences in the fasting glucose and fasting insulin levels between the CTL 100 and CTL 300 groups (*p*<0.05) (Table 3).

**Table 3:** Levels of glucose, insulin, glucagon, and leptin in the C57BL/6J-*db/db* mice with dietary supplementation of *Cudrania tricuspidata* water extract

		C57BL/6J-*db/db* mice		
		
	Normal control	Control	CTL 100	CTL 300
Fasting glucose (mg/dL)	115.0±18.0^c^	490.3±49.1^a^	409.7±36.6^b^	363.0±46.4^b^
Fasting insulin (ng/mL)	0.69±0.15^c^	6.55±0.53^a^	4.97±0.70^b^	4.50±0.69^b^
HOMA-IR^1^	0.28±0.06^c^	11.21±1.27^a^	7.07±0.76^b^	5.74±1.29^b^
Glucagon (ng/mL)	0.33±0.08^ns^	0.42±0.06	0.44±0.05	0.41±0.02
Insulin/glucagon	2.26±0.95^c^	15.85±1.99^a^	11.30±1.36^b^	10.94±1.73^b^
Leptin (ng/mL)	12.2±5.7^c^	131.4±21.0^a^	108.6±13.3^b^	99.6±12.9^b^

1 HOMA-IR = fasting glucose (mmol/L)×fasting insulin (µIU/mL)/22.5.

HOMA-IR, homeostasis model assessment-estimated insulin resistance; CTL, water extract from *C. tricuspidata* leaves. All data are expressed as mean±standard deviation (*n*=8). Different letters show a significant difference at *p*<0.05 as determined by Duncan's multiple range test.

There was no statistically significant difference among any of the groups in the level of glucagon. Consequently, the insulin/glucagon ratio was statistically identical to the ratio with fasting insulin. The level of leptin was significantly increased in the C57BL/6J-*db/db* control group compared with the normal control group. The level of leptin was significantly decreased in the CTL dietary supplement groups compared with that in the C57BL/6J-*db/db* control group, but there was no marked difference in this parameter between the CTL 100 and CTL 300 groups (*p*<0.05) (Table 3).

### Effect of CTL on lipid profiles in the C57BL/6J-*db/db* mice

The effect of CTL on the lipid profiles of the C57BL/6J-*db/db* mice is shown in Table 4. The levels of triglyceride, total cholesterol, LDL/VLDL cholesterol, and HDL cholesterol increased significantly in the C57BL/6J-*db/db* control group compared with those of the normal control group. The levels of triglyceride and LDL/VLDL cholesterol in the C57BL/6J-*db/db* mice fed the CTL dietary supplement declined significantly in a dose-dependent manner. The levels of HDL cholesterol and total cholesterol were significantly decreased in the CTL 300 group when compared with those of the C57BL/6J-*db/db* control group. However, the levels of HDL cholesterol and total cholesterol in the CTL 100 group showed no significant difference when compared with the C57BL/6J-*db/db* control group (*p*<0.05) (Table 4).

**Table 4:** Lipid profiles, AI, and HTR in the C57BL/6J-*db/db* mice with dietary supplementation of *Cudrania tricuspidata* water extract

		C57BL/6J-*db/db* mice		
		
	Normal control	Control	CTL 100	CTL 300
Triglyceride (mM)	1.73±0.18^d^	4.22±0.45^a^	3.14±0.35^b^	2.61±0.42^c^
Total cholesterol (mg/dL)	70.6±8.9^c^	120.5±11.3^a^	115.6±3.9^a^	95.6±10.5^b^
LDL/VLDL cholesterol (mg/dL)	9.05±1.92^d^	29.99±6.20^a^	22.44±2.30^b^	16.23±3.36^c^
HDL cholesterol (mg/dL)	42.97±3.46^b^	59.05±1.67^a^	61.49±2.54^a^	60.19±3.38^a^
AI^1^	0.64±0.15^b^	1.04±0.19^a^	0.88±0.11^a^	0.59±0.17^b^
HTR (%)^2^	61.33±5.84^a^	49.29±4.23^b^	53.23±3.06^b^	63.47±6.65^a^

1 AI (atherogenic index)=(total cholesterol – HDL cholesterol)/HDL cholesterol.

2 HTR (%) = HDL cholesterol/total cholesterol×100.

All data are expressed as mean±standard deviation (*n*=8). Different letters show a significant difference at *p*<0.05 as determined by Duncan's multiple range test.

Compared with the atherogenic index (AI) and the HDL cholesterol/total cholesterol ratio (HTR) of the normal control group, the AI was significantly elevated in the C57BL/6J-*db/db* control group and the HTR was significantly decreased. There were no significant differences in these parameters in the CTL 100 group compared with those in the C57BL/6J-*db/db* control group. However, there was a significant decrease in the AI and a significant increase in the HTR of the CTL 300 group compared with those in the C57BL/6J-*db/db* control group (*p*<0.05) (Table 4).

### Effect of CTL on histological observation in the liver and the fat of the C57BL/6J-*db/db* mice

**Fig. 2:**
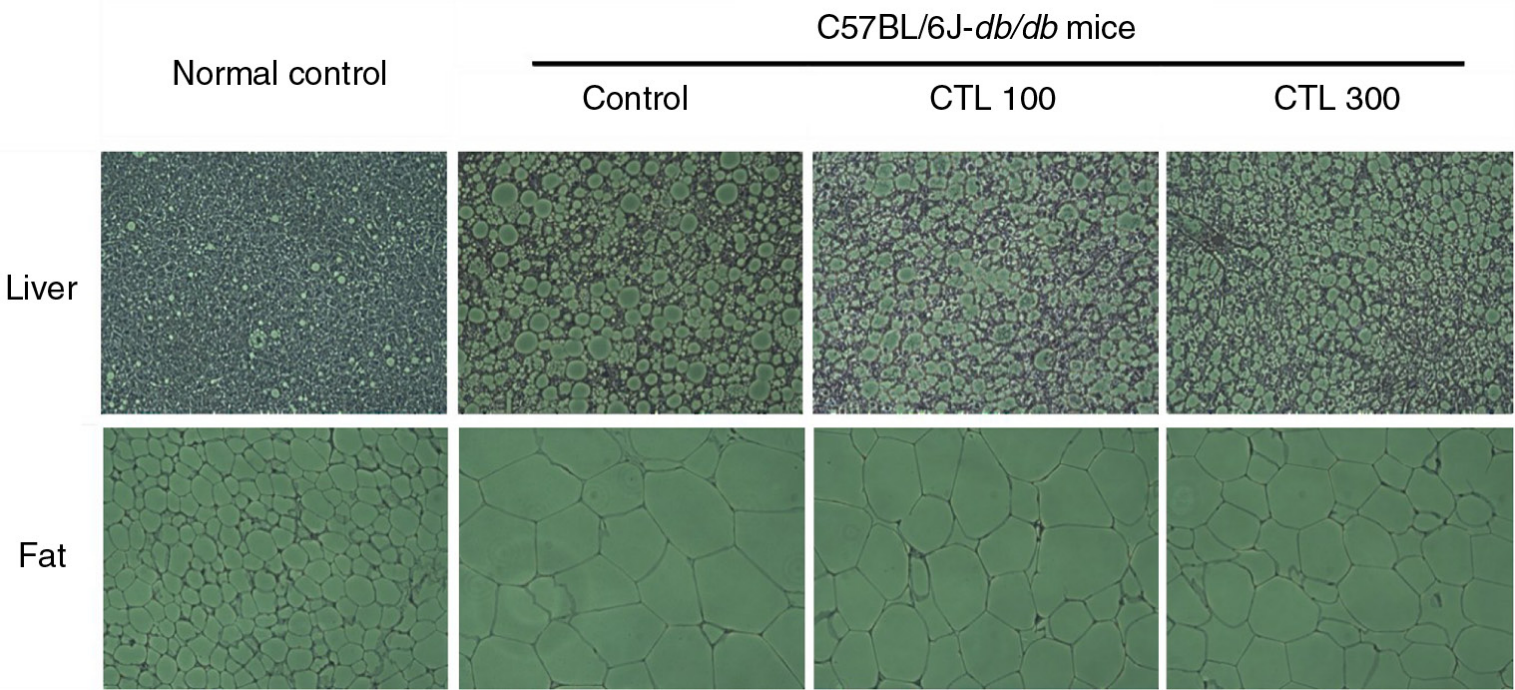
Representative images of histological observation in the liver and the fat of C57BL/6J-*db/db* mice with dietary supplementation of *Cudrania tricuspidata* water extract.

We examined the histology of abdominal fat and the liver by staining with hematoxylin and eosin (H&E). As shown in Fig. 2, the abdominal fat and liver of the C57BL/6J-*db/db* mice contained large lipid droplets, which were not observed in the normal mice. Histological changes and the accumulation of large lipid droplets observed in the C57BL/6J*db/db* control group were attenuated in the CTL 300 group.

### Effect of CTL on ER stress in the livers of C57BL/6J-*db/db* mice

**Fig. 3:**
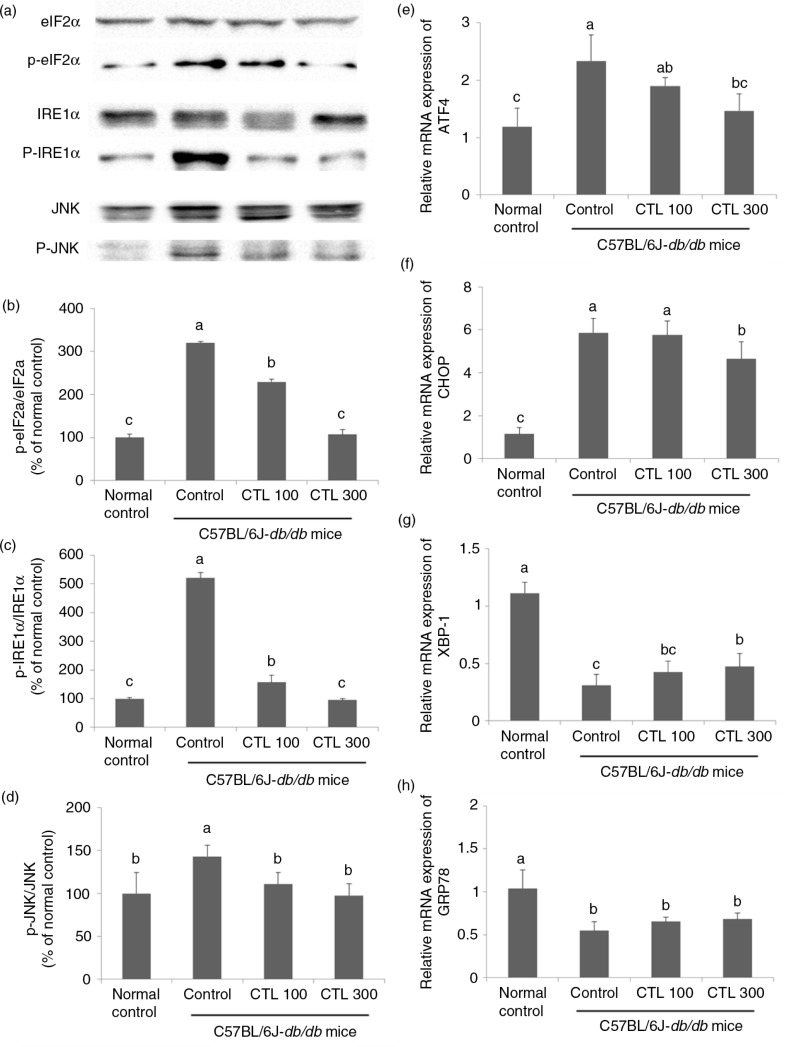
Effect of dietary supplementation with *Cudrania tricuspidata* water extract on endoplasmic reticulum stress in the livers of C57BL/6J-*db/db* mice. (a) Representative Western blots for total protein and phosphorylate expression of eIF2α, JNK, and IRE-1α in the livers of the C57BL/6J-*db/db* mice. Densitometric analysis of phosphorylate expression of (b) eIF2α, (c) JNK, and (d) IRE-1α. mRNA expression of (e) ATF4, (f) CHOP, (g) XBP-1, and (h) GRP78 in the livers of the C57BL/6J-*db/db* mice. Data are expressed as mean±standard deviation (*n*=4). Different letters show a significant difference at *p*<0.05 as determined by Duncan's multiple range test.

The phosphorylation of eIF2α, IRE1α, and JNK was significantly increased in the livers of the C57BL/6J-*db/db* control group compared with those of the normal control group. In the CTL dietary supplement groups, the phosphorylation of eIF2α, IRE1α, and JNK were significantly decreased compared with the C57BL/6J-*db/db* control group (*p*<0.05) (Fig. 3a–d).

Compared with the normal control group, the levels of mRNA expression of activating transcription factor 4 (ATF4) and C/EBP homologous protein (CHOP) were significantly elevated in the livers of the C57BL/6J-*db/db* control group. The levels of mRNA expression of ATF4 and CHOP were significantly decreased in the CTL 300 group compared with those of the C57BL/6J-*db/db* control group (*p*<0.05) (Fig. 3e and f).

The expression of XBP-1 in the C57BL/6J-*db/db* control group was significantly decreased compared with that in the normal control group. The level of mRNA expression of XBP-1 in the CTL 300 C57BL/6J-*db/db* mice was significantly increased compared with that in the C57BL/6J-*db/db* control group. The level of mRNA expression of XBP-1 in the livers of the CTL 100 group was not significantly different to that in the C57BL/6J-*db/db* control group (*p*<0.05) (Fig. 3g).

The level of mRNA expression of GRP78 in the livers of the C57BL/6J-*db/db* control group was significantly decreased compared with that of the normal control group. There was no significant difference in this parameter between the C57BL/6J-*db/db* control group and the CTL dietary supplement groups (*p*<0.05) (Fig. 3h).

### Effect of CTL on inflammation in the livers of the C57BL/6J-*db/db* mice

**Fig. 4:**
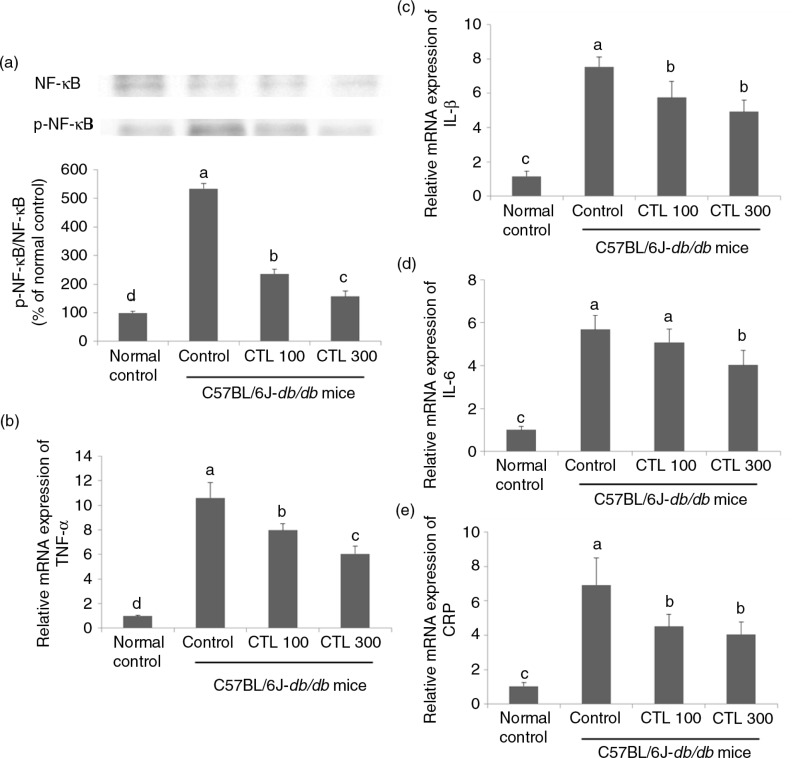
Effect of dietary supplementation with *Cudrania tricuspidata* water extract on inflammation in the livers of C57BL/6J-*db/db* mice. (a) Representative Western blots for total protein and phosphorylate expression of NF-κB in the livers of the C57BL/6J-*db/db* mice. mRNA expression of (b) TNF-α, (c) IL-1β, (d) IL-6, and (e) CRP in the livers of the C57BL/6J-*db/db* mice. Data are expressed as mean±standard deviation (*n*=4). Different letters show a significant difference at *p*<0.05 as determined by Duncan's multiple range test.

The expression of NF-κB phosphorylation was markedly increased in the C57BL/6J-*db/db* mice compared with that in the normal mice. The expression of NF-κB phosphorylation was increased significantly in the C57BL/6J-*db/db* control group compared with that in the normal control group. The dietary supplementation with CTL resulted in a significant decrease in the expression of NF-κB phosphorylation in a dose-dependent manner compared with that in the C57BL/6J-*db/db* control group (*p*<0.05) (Fig. 4a).

There were significant increases in the mRNA expression of the pro-inflammatory cytokines (TNF-α, IL-1β, and IL-6) in the C57BL/6J-*db/db* control group compared with the normal control group. The mRNA expression of TNF-α significantly decreased in a dose-dependent manner in the CTL dietary supplement groups compared with that in the C57BL/6J-*db/db* control group. Compared with the C57BL/6J-*db/db* control group, the mRNA expression of IL-1β was significantly decreased in the CTL dietary supplement groups. The mRNA expression of IL-1β in the CTL 100 and CTL 300 groups did not differ significantly. The level of IL-6 mRNA expression was significantly decreased in the CTL 300 group compared with that in the C57BL/6J-*db/db* control group, whereas there was no significant difference in this parameter in the CTL group compared with that of the C57BL/6J-*db/db* control group (*p*<0.05) (Fig. 4b–d).

The mRNA expression of CRP was increased in the livers of the C57BL/6J-*db/db* control group compared to that in the normal control group. Dietary supplementation with CTL caused a significant decrease in the mRNA expression of CRP, and this parameter did not differ significantly between the CTL 100 and CTL 300 groups (*p*<0.05) (Fig. 4e).

### Effect of CTL on gluconeogenesis and hepatic insulin resistance in the C57BL/6J-*db/db* mice

**Fig. 5:**
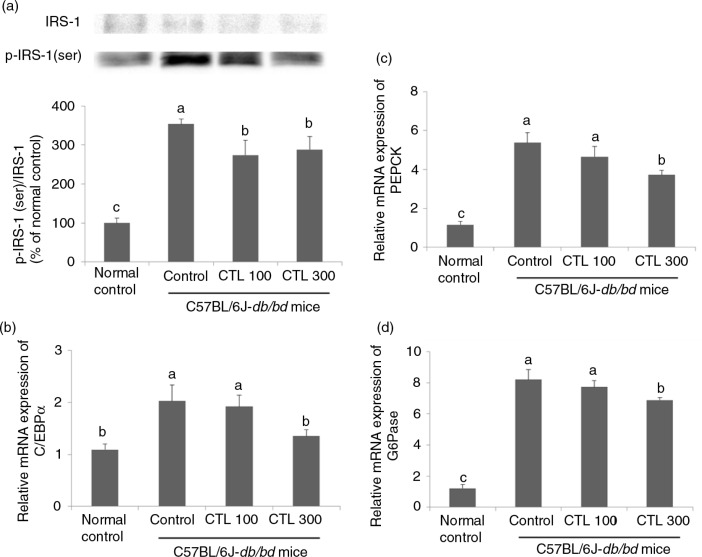
Effect of dietary supplementation of *Cudrania tricuspidata* water extract on gluoconeogenesis and insulin resistance in the livers of C57BL/6J-*db/db* mice. (a) Representative Western blots for total protein and serine phosphorylate expression of IRS-1 in the livers of the C57BL/6J-*db/db* mice. mRNA expression of (b) C/EBPα, (c) PEPCK, and (d) G6Pase in the livers of the C57BL/6J-*db/db* mice. Data are expressed as mean±standard deviation (*n*=4). Different letters show a significant difference at *p*<0.05 as determined by Duncan's multiple range test.

The expression of IRS-1 serine phosphorylation in the livers of the C57BL/6J-*db/db* mice increased significantly when compared with that of the normal mice. There was a significant increase in the expression of IRS-1 serine phosphorylation in the C57BL/6J-*db/db* control group compared with that of the normal control. Dietary supplementation with CTL significantly reduced the expression of IRS-1 serine phosphorylation compared with that of the C57BL/6J-*db/db* control group, but there was no marked difference in expression between the CTL 100 and CTL 300 groups (*p*<0.05) (Fig. 5a).

The mRNA expression of C/EBPα in the livers of the C57BL/6J-*db/db* control group increased significantly compared with that of the normal control group. In contrast, dietary supplementation with 300 mg/kg of CTL caused a marked decrease in the expression of C/EBPα in the liver compared with that of the C57BL/6J-*db/db* control group (*p*<0.05) (Fig. 5b).

The level of mRNA expression of PEPCK and G6Pase in the livers of the C57BL/6J-*db/db* control group increased significantly compared with that of the normal control group. The level of mRNA expression of PEPCK and G6Pase significantly decreased in the CTL 300 group compared with that of the C57BL/6J-*db/db* control group. However, there was no significant difference in the mRNA expression of PEPCK and G6Pase in the CTL 100 group compared with that in the C57BL/6J-*db/db* control group (*p*<0.05) (Fig. 5c and d).

## Discussion

Chronic excess energy intake leads to obesity, which can play a role in the development of hepatic insulin resistance. Although the molecular mechanism of the association between obesity and hepatic insulin resistance is unclear, it has been reported that obesity leads to hepatic ER stress and inflammation, which can induce the development of insulin resistance in several tissues (11, 12, 16). Under chronic excess energy intake conditions, the secretion of free fatty acids, leptin, and pro-inflammatory cytokines, including TNF-α and IL-6, may increase in white adipose tissue. In addition, the blood glucose and insulin level can also increase under chronic excess energy intake conditions, resulting in inflammation and the accumulation of unfolded proteins in the ER lumen of the liver (32, 33).

The accumulation of unfolded proteins triggers the unfolded protein response for the degradation of unfolded proteins or the expression of chaperone proteins to help with protein folding. However, insufficient reduction of unfolded proteins or aggravation of the accumulation of unfolded proteins can induce ER stress and cell death (34). ER stress is triggered by three main signaling systems mediated by ER transmembrane proteins – PERK, ATF6, and IRE1α. Autophosphorylation of PERK phosphorylates the eukaryotic initiation factor 2 α subunit (eIF2α), which can reduce the workload of the ER by reducing the accumulation of unfolded proteins (35, 36). However, when phosphorylation of eIF2α leads to the translation of ATF4, the translation can induce the apoptotic pathways mediated by the activation of CHOP, a proapoptotic transcription factor (37). In addition, some studies have reported that continuous phosphorylation of eIF2α promoted C/EBPs translation, which subsequently induced gluconeogenic gene expression (38–40). Pedersen et al. demonstrated that the C/EBPα transcription factor regulates hepatic glucose metabolism upregulation of the hepatic gluconeogenic G6Pase and PEPCK (40).

Phosphorylation of IRE1α initiates the splicing of the X-box binding protein 1 (XBP-1) mRNA, which binds to ERSE (ER stress–response element) and activates the expression of chaperone proteins (41). On the other hand, phosphorylation of IRE1α under ER stress conditions phosphorylates JNK, which is known to be activated under diabetic conditions and associated with the development of insulin resistance (42, 43). Nakatani et al. reported that suppression of the JNK pathway decreased hepatic insulin resistance and improved glucose tolerance in diabetic animal models (43).

In the present study, we used a C57BL/6J-*db/db* mouse model to investigate the associations among hepatic insulin resistance, ER stress, and inflammation in obesity. The C57BL/6J-*db/db* mouse is a genetic animal model of obesity and type 2 diabetes and is a useful model to study the pathogenesis of obesity-induced insulin resistance when leptin receptor activity is deficient (44, 45). The present study revealed marked increases in the weight gain, blood insulin, blood lipids, and levels of blood glucose during OGTTs in the C57BL/6J-*db/db* mice compared with those of the normal mice. In addition, we found that the ER stress response was activated in the livers of the C57BL/6J-*db/db* mice in response to increases in the phosphorylation of eIF2α IRE1α, JNK, and IRS-1 serine and increases in the mRNA expression of ATF4, C/EBPα, and CHOP. However, the expression of XBP-1 and the ER chaperone protein (GRP78) decreased in the livers of the C57BL/6J-*db/db* mice.

A report by Achard et al. revealed that saturated fatty acid exposure induced severe ER stress in a dose-dependent manner, contributing to insulin resistance by decreasing the phosphorylation of Akt and glycogen synthesis and increasing the expression of G6Pase (46). Another report indicated that the expression of ER chaperone proteins was reduced in the livers of diabetic *db/db* mice and that the IRE1/XBP-1 pathway was required for the folding of unfolded proteins in the ER through the expression of ER chaperone protein (47). Ozcan et al. reported that chemical or pharmaceutical chaperones such as 4-phenylbutyric acid enhanced the folding capacity of unfolded proteins in the ER and improved systemic insulin action in *ob/ob* mice (48). These results and those of the present study indicate that hepatic ER stress in obesity may play a role in hepatic insulin resistance.

We investigated the molecular mechanisms of CTL that contribute to ameliorated insulin resistance in C57BL/6J-*db/db* mice. We found a marked decrease in the activation of the hepatic ER stress response and a decrease in the phosphorylation of eIF2α, IRE1α, JNK, and IRS-1 serine, as well as in the mRNA expression of ATF4, C/EBPα, and CHOP in the livers of the C57BL/6J-*db/db* mice treated with CTL. However, we found no significant difference in the level of GRP78 mRNA expression in the dietary supplement groups compared with that of the C57BL/6J*db/db* control group. Overall, these results suggest that CTL can improve hepatic insulin resistance and hyperglycemia by suppressing obesity-induced ER stress but that it does not affect the expression of chaperone proteins.

Many recent studies have documented the association between the chronic low-grade inflammation of white adipose tissue and the development of insulin resistance in several tissues under chronic obesity conditions (49, 50). Xu et al. found that inflammatory responses by macrophages in the white adipose tissue of mouse models for genetic and high-fat diet-induced obesity contributed to the pathogenesis of insulin resistance (49). In addition, Kern et al. found that secretion and expression of IL-6 and TNF was significantly higher in obese subjects (BMI 30–40 kg/m^2^) compared with lean subjects (BMI<25 kg/m^2^) (50). Blood environment such as increased cytokines and leptin under chronic obesity conditions can stimulate inflammation through the NF-κB and JNK pathways in the liver (32, 33). It was reported that an increase in hepatic production of pro-inflammatory cytokines, including IL-6, IL-1β, and TNF-α, through NF-κB activation was found in a group of mice fed a high-fat diet (22).

The present study revealed significant increases in the expression of pro-inflammatory cytokines as well as CRP and NF-κB phosphorylation in the C57BL/6J-*db/db* control group compared to those in the normal control group. Interestingly, we showed that dietary supplementation with CTL induced a statistically significant decrease in the expression of pro-inflammatory cytokines as well as CRP and NF-κB phosphorylation. In a study by Jeong et al., xanthone isolated from CT suppressed the production of TNF-α and IL-1β and the degradation of I kappa B-α in RAW264.7 macrophages (51). Joo et al. showed that CT glycoproteins suppressed the expression of inflammatory-related proteins through regulation of the NF-κB pathway (27). According to these reports and our present results, we suggest that CTL could improve insulin resistance by suppressing inflammation and inhibiting the activation of NF-κB and JNK signaling in the liver induced by obesity.

Guo et al. reported that pathogenic ER stress leads to inflammation via NF-κB and JNK activation, promoting inflammatory cytokine production and that the ER stress-induced inflammation is directly responsible for the pathogenesis of several metabolic and inflammatory diseases (52). On the other hand, it was reported that exposure to TNF-α-induced ER stress itself can lead to the expression of TNF-α and inflammatory responses (24). Thus, the inflammatory mediators can also induce ER stress.

## Conclusions

Our findings demonstrate that chronic obesity-induced insulin resistance is associated with inflammation-induced ER stress and ER stress-induced inflammation directly or indirectly. Taken together, we found in the present study that obesity induced hepatic insulin resistance through ER stress and inflammation and also that CT exerted antihyperglycemic effects by suppressing ER stress and inflammation in C57BL/6J-*db/db* mice.
